# Alternative Complement Pathway Inhibition Does Not Abrogate Meningococcal Killing by Serum of Vaccinated Individuals

**DOI:** 10.3389/fimmu.2021.747594

**Published:** 2021-10-08

**Authors:** Emma Ispasanie, Lukas Muri, Anna Schubart, Christine Thorburn, Natasa Zamurovic, Thomas Holbro, Michael Kammüller, Gerd Pluschke

**Affiliations:** ^1^ Molecular Immunology Unit, Swiss Tropical and Public Health Institute, Basel, Switzerland; ^2^ University of Basel, Basel, Switzerland; ^3^ Translational Medicine-Preclinical Safety, Novartis Institutes for Biomedical Research, Basel, Switzerland; ^4^ Global Drug Development, Novartis Pharma AG, London, United Kingdom; ^5^ Global Drug Development, Novartis Pharma AG, Basel, Switzerland

**Keywords:** *Neisseria meningitidis*, alternative pathway, vaccination, immunotherapy, complement inhibitor

## Abstract

Dysregulation of complement activation causes a number of diseases, including paroxysmal nocturnal hemoglobinuria and atypical hemolytic uremic syndrome. These conditions can be treated with monoclonal antibodies (mAbs) that bind to the complement component C5 and prevent formation of the membrane attack complex (MAC). While MAC is involved in uncontrolled lysis of erythrocytes in these patients, it is also required for serum bactericidal activity (SBA), i.e. clearance of encapsulated bacteria. Therefore, terminal complement blockage in these patients increases the risk of invasive disease by *Neisseria meningitidis* more than 1000-fold compared to the general population, despite obligatory vaccination. It is assumed that alternative instead of terminal pathway inhibition reduces the risk of meningococcal disease in vaccinated individuals. To address this, we investigated the SBA with alternative pathway inhibitors. Serum was collected from adults before and after vaccination with a meningococcal serogroup A, C, W, Y capsule conjugate vaccine and tested for meningococcal killing in the presence of factor B and D, C3, C5 and MASP-2 inhibitors. B meningococci were not included in this study since the immune response against protein-based vaccines is more complex. Unsurprisingly, inhibition of C5 abrogated killing of meningococci by all sera. In contrast, both factor B and D inhibitors affected meningococcal killing in sera from individuals with low, but not with high bactericidal anti-capsular titers. While the anti-MASP-2 mAb did not impair SBA, inhibition of C3 impeded meningococcal killing in most, but not in all sera. These data provide evidence that vaccination can provide protection against invasive meningococcal disease in patients treated with alternative pathway inhibitors.

## 1 Introduction

*Neisseria meningitidis* is a Gram-negative bacterium, also called meningococcus, which asymptomatically colonizes the nasopharyngeal mucosa of 5-10% of the adult population. Colonization mostly induces immunity with protective antibody response, but in rare cases the meningococci can gain access to the circulation and may cause systemic disease such as sepsis and meningitis (1–3). Based on the chemical composition of the capsular polysaccharides, meningococci are divided into 12 serogroups – six of which are reported to cause invasive disease (4, 5). As no immunological memory formation and hyporesponsiveness upon repeated vaccination with unconjugated meningococcal vaccines have been found (6–8), vaccination with conjugated meningococcal capsule vaccines (e.g. MenACWY-TT and MenACWY-CRM_197_) for serogroups A, C, W and Y and with a protein-based vaccine for B meningococci is currently recommended (5).

Killing of *N. meningitidis* by the complement system membrane attack complex (MAC)-induced bacteriolysis represents a key element of host immune defense against meningococci. Determination of the serum bactericidal antibody activity (SBA) serves as surrogate of meningococcal vaccine efficacy as it measures the MAC-induced killing (9). The SBA assay measures classical pathway (CP) complement-mediated killing by circulating antibodies that induce lysis of meningococci. The dilution of a test serum that results in at least a 50% decrease in colony forming units (CFUs) is defined as a bactericidal titer (10). SBA assays require active intrinsic or exogenous complement, and for serogroup C meningococci SBA titers of either ≥4 or ≥8 have been established as correlates of protection when using human or rabbit complement, respectively (11, 12). Antibody-mediated activation of the CP depends on factors like subclass and avidity of antibody binding, and the accessibility, density and distribution of the target antigen (13, 14). Binding of the C1 complex to the Fc region of the antibodies, which are bound to their target antigen on the bacterial cell surface, initiates CP activation by cleavage of C4 and covalent C4b deposition on the meningococcal cell surface. C4b then recruits C2, which is also cleaved by C1s. The C4b2b complex is a C3 convertase, which cleaves C3 into C3a and C3b, and the generated C3b binds to C4b2b to form a C5 convertase (C4b2b3b). The C5 convertase then cleaves C5 into C5a and C5b. Subsequent interactions between C5b and the other terminal components C6, C7, C8, and C9 form the MAC (C5b-9), which lyses the meningococcal cell by forming pores on the membranes (15, 16).

In the absence of antibodies, alternative complement pathway (AP) activation can also lead to C3b deposition on the meningococcal cell surface, and it also amplifies complement activation initiated by either complement pathway. While the bactericidal efficacy of antibodies directed against abundantly expressed surface antigens like the capsular polysaccharide seems to be unaffected by AP inhibition, antibodies directed against sparsely expressed surface antigens are more dependent on amplification by the AP (17). Activation of the AP can occur directly by C3b or is mediated by the hydrolysis of C3 resulting in the formation of C3(H_2_O) that leads to factor B (fB) binding and cleavage of bound fB by factor D (fD) (18). The resulting C3(H_2_O)Bb or C3bBb complexes have C3 convertase activity. The importance of the AP for defense against meningococci is reflected by a strong association of invasive meningococcal disease with deficiencies in fD (19–21) and fB (22, 23). Moreover, deficiency in properdin, which stabilizes the AP C3-convertase C3bBb, is associated with a high risk for severe meningococcal disease (24–27) and terminal complement component-deficient individuals have a 5000- to 10 000-fold greater risk of contracting meningococcal diseases (28). Eculizumab, a humanized monoclonal antibody (mAb) is being used to treat patients suffering from atypical hemolytic uremic syndrome (aHUS) and paroxysmal nocturnal hemoglobinuria (PNH). Such individuals have an excessive over-activation of their complement system due to either inherited or acquired genetic mutations or autoantibodies contributing to the pathogenesis. Eculizumab binds C5 and prevents C5 cleavage, which is necessary for the formation of the MAC. By limiting the excessive activation of complement in PNH and aHUS individuals, Eculizumab has proven successful for the treatment of these patients. However, use of Eculizumab is associated with a 1000-2000 fold increased incidence of meningococcal disease, and even after vaccination susceptibility to infection remains substantial (29, 30). Anti-C5 antibodies have black box warnings reflecting their higher infection rate. Currently, several promising drug candidates targeting various other complement proteins in the cascade are in development for the treatment of PNH, aHUS and other complement disorders (16). Here we have investigated the survival of *N. meningitidis* in sera from individuals before and after vaccination with a conjugate MenACWY vaccine, and together with several drug candidates inhibiting complement components factor B, factor D, C3, C5 and MASP-2.

## 2 Methods

### 2.1 Ethical Approval

Ethical approval was obtained from the Ethical Committee of Northwest and Central Switzerland (Ethikkommission Nordwest- und Zentralschweiz (EKNZ), Studie 2018-02341).

### 2.2 Bacterial Isolates

*Neisseria meningitidis* isolates were obtained from a longitudinal meningococcal meningitis study (31) and from the Institute for Infectious Diseases (University of Bern) (**Table 1**).

**Table 1:** *Neisseria meningitidis* isolates used in this study.

Serogroup	Isolate	Origin	Year of isolation	MLST*	Source
A	2602	Burkina Faso	2007	2859	case
C	4207	Switzerland	2009	12511	case
W	1682	Accra, Ghana	2003	11	case
Y	5810	Switzerland	2015	23	case

*Multi-locus sequence typing (MLST).

### 2.3 Human Sera

Five healthy volunteers were recruited upon informed and written consent. Four (2, 3, 9 and 12) had been immunized with meningococcal vaccines prior to enrolment (**Table 2**). All five received one dose of the MenACWY-CRM_197_ vaccine (Menveo^®^, GSK) in 2019-2020, in the framework of this study. Sera from venous blood were taken before, 2 weeks and 2 months after this vaccination. Vacuette^®^ CAT serum tubes (Greiner Bio-one) were used for collection of sera and preservation of their terminal complement activity was reconfirmed (Complement TCC, Svar). The numbers of the subjects are not consecutive, as these volunteers were part of a cohort receiving different vaccines. Subjects included in this study are not reflecting a selection, but include all subjects receiving a Menveo vaccination.

**Table 2:** Description of the subjects and their vaccination status prior to study initiation.

Subject number	Age	Gender	Previous meningococcal vaccinations (year)
2	31	F	Menveo (2015, 2011) NeisVac-C^†^ (2007)
3	66	M	Menveo (2014) Mencevax* (2006) MenA+C^‡^ (1996, 1997)
9	32	M	NeisVac-C (2007)
11	43	F	None
12	29	M	Menveo (2013) NeisVac-C (2008)

*Unconjugated MenACWY vaccine, Mencevax™, Pfizer.

^†^Conjugate MenC vaccine, NeisVac-C, Pfizer.

^‡^Unconjugated meningococcal polysaccharide A + C vaccine, Sanofi Pasteur.

### 2.4 Serological Analysis

#### 2.4.1 Serogroup-Specific IgG and IgM Antibody Concentrations

Sera were tested using an ELISA assay optimized by Holder et al., Joseph et al. and Findlow et al. (32–34) In short, Maxisorp 96-well plates (Nunc) were coated over-night at 4°C with polysaccharide (PS). PS was mixed with methylated human serum albumin (mHSA, NIBSC 12/176) at following concentrations for A (NIBSC 13/267) and C (NIBSC 07/318): 5 µg/mL PS and 5 µg/mL mHSA; for W (NIBSC 01/428) and Y (NIBSC 01/426): 1 µg/mL PS and 2 µg/mL mHSA. The anti-meningococcal serum CDC 1992 was used as reference with assigned concentrations of serogroup-specific antibodies (NIBSC 99/706). After over-night serum incubation at 4°C, plates were incubated for 2.5 hours at room temperature with goat anti-human IgG H+L HRP (1:3000, BioRad) and mouse anti-human IgM HRP (1:5000, Southern Biotech) antibodies. Following addition of KPL peroxidase (SeraCare) and 0.5M sulphuric acid, absorbance was read at 450nm.

#### 2.4.2 SBA Assay

Serum bactericidal titers were measured as previously described (35). Each reaction mixture contained approximately 400 CFUs of mid-log phase bacteria grown in Frantz medium containing 2mM cytidine-5′-monophospho-N-acetylneuraminic acid (CMP-NANA, Sigma-Aldrich) and 20% human serum with internal complement preserved (36). Bactericidal titers were defined as the interpolated dilution of serum resulting in 50% killing of bacteria after 60 minutes incubation at 37°C compared to the mean CFUs in the control reactions at time 0 (11, 37). Antibody titers were log10-transformed using GraphPad Prism v.8.2.1, and concentrations of <5 were assigned the value 2.5 (100% survival).

#### 2.4.3 Survival of Meningococci in Presence of Human Serum and Inhibitors

Inhibitors tested included factor B (Iptacopan, LNP023, Novartis) (38), factor D (CMS487, Novartis) (38), C3 (CP-40, Bachem), anti-C5 antibody (Tesidolumab, LFG316) (39, 40) and anti-MASP-2 antibody (Narsoplimab) (41, 42). The inhibitors were added to the SBA reaction mixture. The percent survival after incubation at 37°C for 60 minutes was assessed by plating on chocolate agar (Biomerieux) and compared to CFUs in the control reactions at time 0. SBA analyses with the anti-C5 inhibitor in combination with other inhibitors was not performed, as the C5 inhibitor alone already blocks the terminal pathway completely.

### 2.5 Statistical Analysis

The correlation between IgG concentration and bactericidal activity was assessed using Spearman’s correlation coefficient test. The effect of inhibitors on the survival of meningococci in post- and pre-vaccination sera was evaluated using two-way ANOVA and Tukey’s multiple comparisons test.

### 2.6 Data Sharing Statement

The data that support the findings of this study are available from the corresponding author upon request.

## 3 Results

### 3.1 Anti-Capsular Antibody Response and SBA Titers Elicited by Vaccination With Menveo

The aim of the study was to investigate the effect of complement inhibitors on SBA against meningococci in naïve and vaccinated individuals. As a first step, we analyzed the correlation between serum anti-capsular antibody concentrations and SBA titers. In order to have sera with a broad range of antibody concentrations available for the analysis, five adult subjects with different histories of previous meningococcal vaccinations (**Table 2**) were vaccinated with the conjugate polysaccharide vaccine MenACWY-CRM_197_ (Menveo). Sera were collected prior to vaccination, 2 weeks and 2 months post-vaccination.

**Figure 1:**
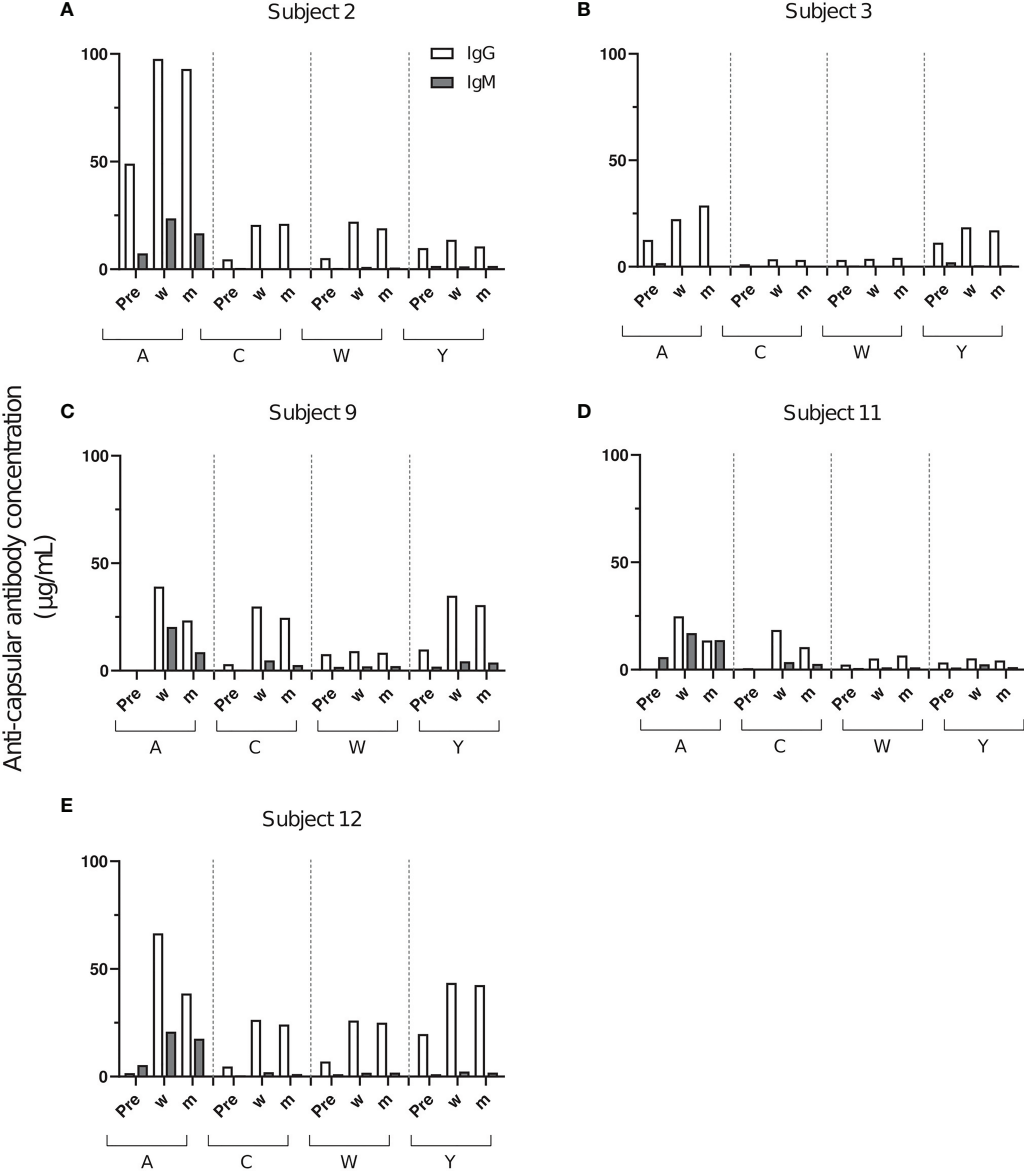
Concentrations of anti-capsular IgG and IgM after vaccination with Menveo. Serum samples were taken before vaccination (Pre), 2 weeks (w) and 2 months post-vaccination (m) from five healthy volunteers **(A–E)**. Each bar represents the results of two measurements against A, C, W and Y polysaccharide.

Serum anti-capsular polysaccharide antibody concentrations were determined by ELISA (**Supplementary Table 1**). All subjects had measurable IgG titers against several capsule polysaccharides prior to Menveo vaccination (**Figure 1**). Overall, a wide variation in individual response patterns was observed after vaccination. In most cases, anti-capsular IgG reached the highest concentration 2 weeks after vaccination and declined thereafter (**Figure 1**). In all five subjects, the serogroup A polysaccharide specific IgG antibodies had the highest IgG concentrations, and IgM was primarily found against this serogroup. In some cases, only very low anti-capsular IgG concentrations were elicited (i. e. antibody responses against C and W in subject 3, against C in subject 9 and against W and Y in subject 11, **Figures 1B–D**).

**Figure 2:**
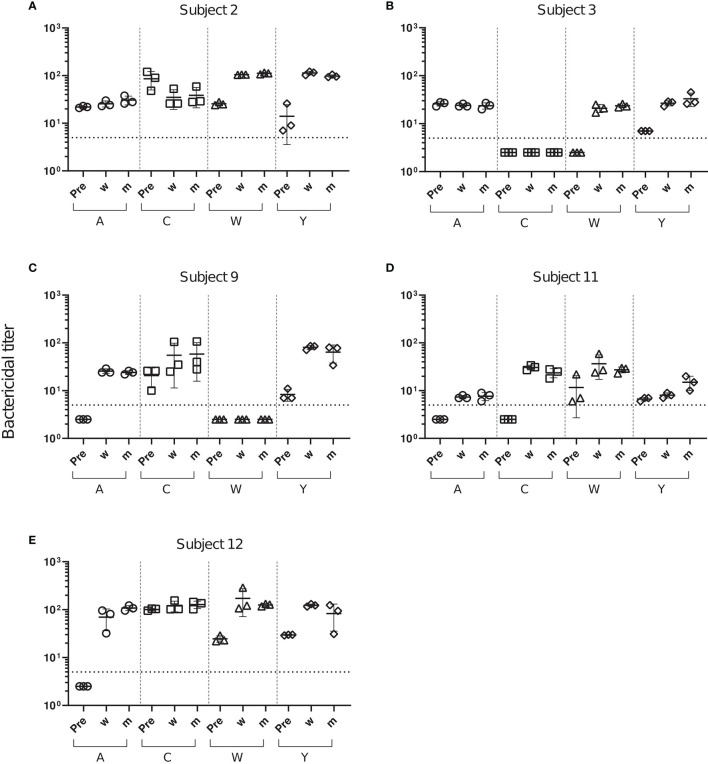
Changes in SBA titers against meningococcal case isolates after Menveo vaccination. SBA titers of sera from five subjects **(A–E)** taken prior to vaccination (Pre), 2 weeks (w) and 2 months (m) after vaccination with Menveo. Bactericidal titers are the reciprocal dilutions of serum that result in 50% killing of the bacteria after 60 minutes incubation. Error bars represent standard deviation of the mean titer of triplicate technical replicates. The bottom dotted line indicates the lowest serum dilution measured (1:5). Symbols below that line set to half of the lowest serum dilution, indicate SBA titers below detection limit.

Changes in SBA titers against meningococcal case isolates were assessed using serum samples from the three sampling time points with internal complement of the test sera preserved. After the Menveo vaccination, five initially SBA negative serum/serogroup combinations (3/W, 9/A, 11/A, 11/C and 12A) showed SBA titers >4, which has been established as a correlate of protection (**Figures 2B–E**). Furthermore, a marked increase in pre-existing SBA titers was observed for 10/20 of the serogroup-serum combinations (**Figure 2** and **Supplementary Table 3**). SBA titers did not change for two (3/A, 12/C) and decreased for one combination (2/C). Only two SBA negative serogroup-serum combinations (3/C and 9/W) remained SBA negative. In most cases, SBA titers of sera taken 2 weeks and 2 months after the vaccination were comparable (**Supplementary Table 2**).

**Figure 3:**
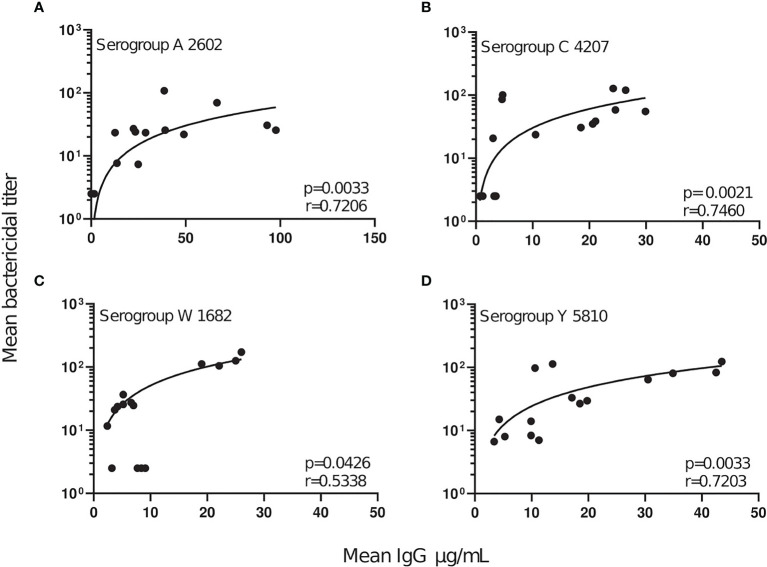
Correlation of SBA titers and serogroup-specific anti-capsular IgG concentrations. For each serogroup tested **(A–D)**, the mean concentration of anti-capsular IgG was plotted against the corresponding mean serogroup-specific SBA titer. A linear regression line was plotted to outline the data points and show the degree of discordance. The correlation between the two non-parametric paired data sets were calculated using GraphPad Prism v.8.2.1, and the Spearman’s correlation coefficient test (r). *P* values of ≤0.05 were considered to be statistically significant.

Analysis of the relationship between the serogroup-specific IgG concentrations and the SBA titers revealed a significant positive correlation between antibody concentration and function (**Figure 3**). For all serogroups significant sharp increases in SBA titers were observed up to an anti-capsular IgG concentration of about 5 µg/mL. At higher IgG concentrations, SBA was further rising only moderately (**Figure 3**). The observed effective IgG concentration correlated well with the prediction of 2 μg/mL as surrogate marker of protection against serogroup A (43).

### 3.2 Effect of Complement Inhibitors on SBA

**Figure 4:**
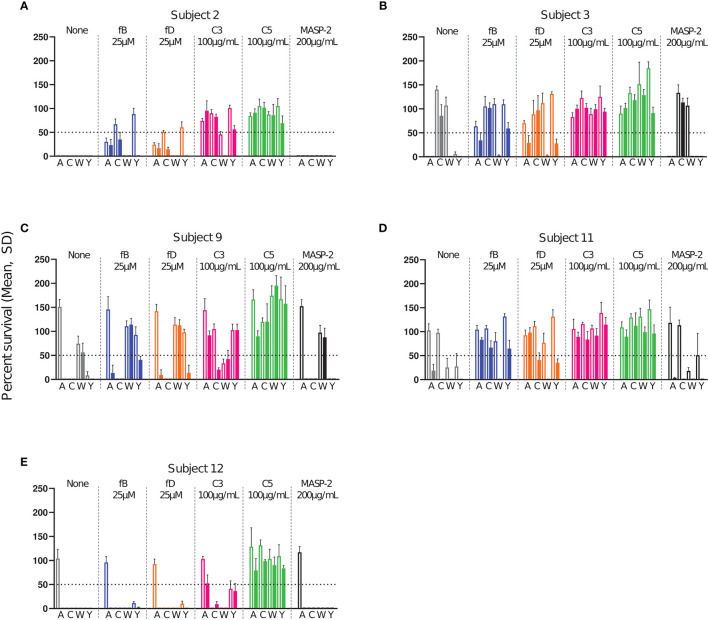
Effect of complement inhibitors on the survival of meningococci in sera from subjects 2 **(A)**, 3 **(B)**, 9 **(C)**, 11 **(D)** and 12 **(E)** taken prior to vaccination and 2 months after vaccination with Menveo. Sera were diluted 1:5 and assayed with the internal complement. Data shown in the graphs are with the highest inhibitor concentration tested, fB: 25 µM, fD: 25 µM, C3: 100 µg/mL, anti-C5 mAb: 100 µg/mL and anti-MASP-2 mAb: 200 µg/mL. In each graph, open bars represent pre-vaccination sera and filled bars sera taken 2 months after vaccination. The horizontal dotted line represents 50% survival of bacteria after 60 min incubation as compared to control wells at time 0. Data for each inhibitor are triplicate technical replicates. SD: standard deviation. fB indicates factor B inhibitor Iptacopan; fD, factor D inhibitor CMS487; C3, C3 inhibitor CP-40; C5, anti-C5 mAb Tesidolumab; MASP-2, anti-MASP-2 mAb Narsoplimab.

Killing of the bacteria in the presence of complement inhibitors was compared to killing without inhibitor. Inhibitor concentrations tested were for factor B inhibitor (Iptacopan) 0.05-25 µM, factor D inhibitor (CMS487) 0.05-25 µM, C3 inhibitor (CP-40) 1-100 µg/mL, anti-C5 antibody (Tesidolumab) 1-100 µg/mL and anti-MASP-2 mAb (Narsoplimab) 1-200 µg/mL. Data for all serum-serogroup combinations are shown in detail in **Supplementary Figures 1–4**. Full inhibitory activity was already reached at concentrations of 1 µM for fB and fD inhibitors and 15 µg/mL for the C3 and C5 inhibitors. Furthermore, results obtained with serum taken 2 weeks and 2 months after the vaccination were comparable (**Supplementary Figures 1–4**). Therefore, data obtained with the highest inhibitor concentration with post-dose sera taken 2 months after vaccination and pre-dose sera are summarized as representative results in **Figure 4**.

As expected, addition of the C5 inhibitor abrogated SBA for all pre- and post-vaccination sera (**Figure 4**). A slightly different picture was observed for the C3 inhibitor. Here bacterial killing was in most cases also strongly inhibited, but both pre- and post-vaccination serum of subject 12 retained full SBA for serogroup C and W bacteria (**Figure 4E**). Furthermore, some SBA (bacterial survival <50%) remained in the presence of the C3 inhibitor for the combinations 2/Y, post-9/C, 9/W and 12/Y (**Figures 4A, C, E**), which all had high, but not outstandingly high SBA titers (**Figure 2**). No SBA inhibition was caused by the anti-MASP-2 mAb.

In the presence of the fB inhibitor, only four of the 20 pre-vaccination combinations (2/W, 9/C, 12/C, 12/W) retained full and two (2/A and 12/Y) partial (i.e. bacterial survival <50%) SBA (**Figures 4A, C, E**). After vaccination, these proportions had increased, and 9 post-vaccination combinations retained full and 8 partial activity. Only 3 post-vaccination combinations thus showed a complete lack of killing in the presence of the fB inhibitor. These included the two combinations 3/C and 9/W, where vaccination had led to no measurable SBA, and 11/A, where vaccination had elicited the lowest SBA titer of all SBA active combinations (**Figures 4B–D**). The pattern observed for the fD inhibitor resembled closely that obtained with the fB inhibitor.

**Figure 5:**
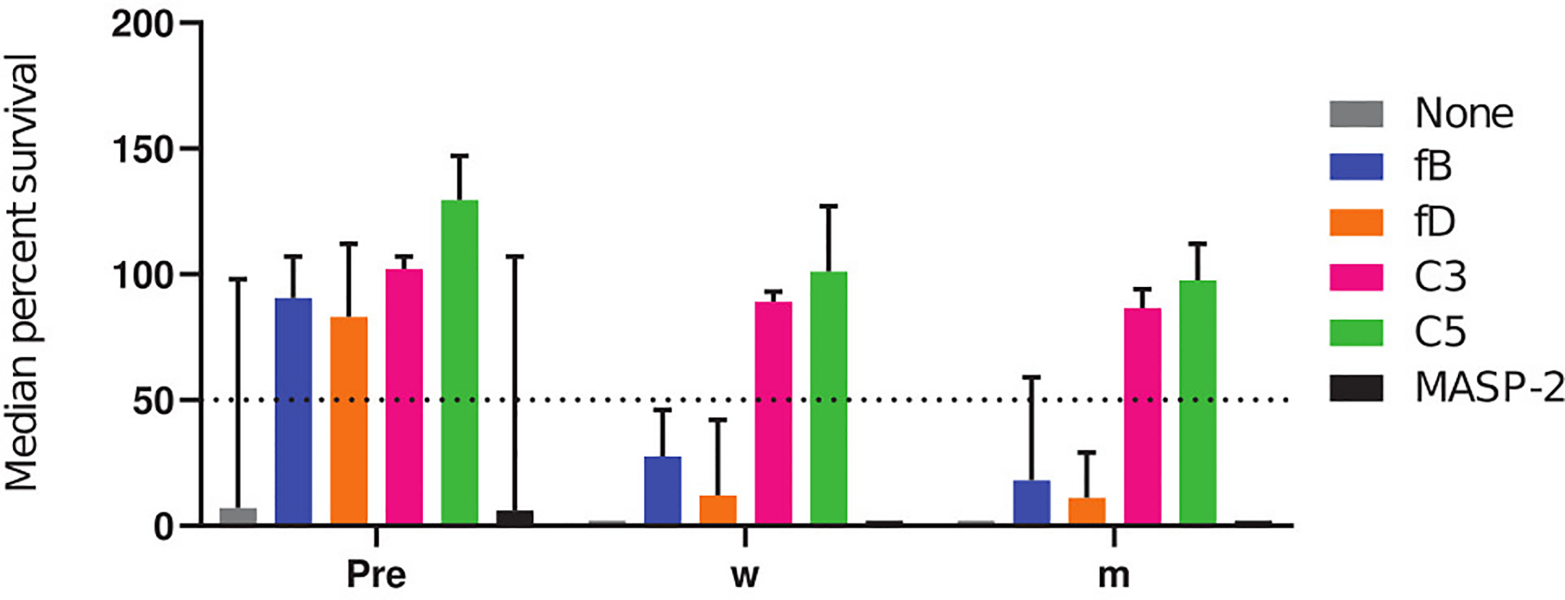
Cumulative comparative analysis of the effect of complement inhibitors on the survival of meningococci in pre- and post-vaccination sera. The percent survival of the three technical replicates of the effect of inhibitors on all meningococcal isolates and subjects were pooled as pre-, 2 weeks (w) and 2 months (m) post-vaccination. The horizontal dotted line represents 50% survival of bacteria after 60 min incubation. fB indicates factor B inhibitor Iptacopan; fD, factor D inhibitor CMS487; C3, C3 inhibitor CP-40; C5, anti-C5 mAb Tesidolumab; MASP-2, anti-MASP-2 mAb Narsoplimab.

To summarize the effect of the different complement inhibitors on the killing of meningococci, the percent survival data for all subjects and serogroups were pooled. Data were grouped into results obtained with pre-vaccination sera and post-vaccination sera collected either 2 weeks (w) or 2 months (m) after vaccination (**Figure 5**). For the AP inhibitors, a significant difference between the pre- and the post-vaccination sera was observed, demonstrating a marked decrease in their effect on SBA after vaccination. In contrast, no significant difference in bacterial survival between the pre- and post-vaccination sera was observed for the C3 and C5 inhibitors. Both inhibitors abrogated meningococcal killing irrespective of the vaccination status (**Figure 5**). Already two weeks after vaccination, antibody concentrations were thus high enough to counteract the inhibition of the SBA by the AP inhibitors.

## 4 Discussion

Treatment with the first complement-targeting drug Eculizumab has shown significant clinical efficacy in patients with PNH and aHUS (15). However, even after meningococcal vaccination, Eculizumab treatment increases susceptibility to invasive meningococcal disease 1000 - 2000-fold (30). As an alternative, a new generation of complement therapeutics, targeting different stages of the complement cascade, is currently in pre-clinical and clinical phases of development. Here we compared the effect of a set of complement inhibitors, including small molecules targeting the AP components fB and fD, a C3 small peptide inhibitor and anti-C5 and anti-MASP-2 mAbs. We investigated the effect of these complement inhibitors on meningococcal killing in the presence of serum from individuals collected before and 2 weeks or 2 months post-immunization with the meningococcal ACWY polysaccharide conjugate vaccine Menveo. After Menveo vaccination, the overall SBA titers against A, C, W and Y meningococci increased for 75% of the serum/serogroup combinations (**Figure 2**), which positively correlated with the increase of anti-capsular IgG concentrations (**Figure 3**). SBA titers increased strongly up to an anti-capsular IgG concentration of about 5 µg/mL. Already 2 weeks after the vaccination, 18/20 serum/serogroup combinations had reached SBA titers >4, which are considered to be protective (11, 44).

SBA against meningococci depends on the formation of the MAC (45) and the clinical use of Eculizumab has confirmed the central role of C5 cleavage in meningococcal immunity. Inhibition of C5 cleavage by Eculizumab prevents the generation of C5b, which is the pivotal step in MAC assembly and meningococcal killing. In addition, lack of release of the pro-inflammatory peptide C5a may also affect meningococcal clearance (29, 35). In our study, anti-C5 mAb at a concentration of 15µg/mL completely abrogated meningococcal killing irrespective of serogroup and vaccination status.

As an alternative to C5 inhibition with Eculizumab, several AP-targeted therapeutics, including the fD inhibitor Danicopan (ACH-4471) (46) and the fB inhibitor Iptacopan (LNP023) (38) are currently being evaluated in the clinic, and successful phase 2 clinical trials with both agents against PNH have been recently reported (47, 48). However, reports that fB and fD deficient individuals are known to present with recurring invasive pneumococcal and meningococcal disease, underscores the importance of the AP in immunity against these bacteria (19, 20). The AP is not only continuously undergoing a low-grade antibody-independent activation, but functions also as an amplifier of C3b deposited through the CP or lectin pathway (LP). While inhibition of C5 abrogated killing of meningococci by all sera, the two AP inhibitors affected meningocccal killing only in sera with low anti-capsular IgG titers. Collated data for all subjects and serogroups tested revealed a difference between the pre- and the post-vaccination sera (**Figure 5**), which was significant, in spite of the fact that vaccination did not elicit in all subjects antibody responses against all serogroups and that some pre-vaccination sera had already SBA titers against certain serogroups. Our data showed, that already two weeks after vaccination, antibody titers were high enough to counteract the inhibition of SBA by the AP inhibitors. Independent of AP amplification, capsule polysaccharide specific antibodies can thus efficiently trigger SBA *via* the CP alone. In accordance with these results, Granoff et al. found that the fD inhibitor ACH-4471 did not affect meningococcal killing by sera of vaccinated subjects in 6/8 meningococcal isolates (35).

The C3 inhibitor CP-40 tested here, is a small peptide analog of the Compstatin family, which is currently under clinical development for the treatment of age-related macular degeneration (49). A PEGylated version of a C3 inhibitor Pegcetacoplan (APL-2) from the same company has now been approved by the FDA as a new treatment for PNH (50–52). C3 inhibition prevents the formation of both C3 and C5 convertases, and accordingly, vaccination did not restore meningococcal killing in the presence of CP-40. However, for some serum/serogroup combinations, CP-40 did not inhibit bacterial killing completely. The inhibitor binds to the native form of C3 and sterically hinders the binding and cleavage of native C3 by C3 convertases (49). However, the C3 inhibitor does not bind or prevent the spontaneous hydrolysis of C3 into fluid phase C3(H_2_O) *via* the “tick-over” process, nor does it inhibit conformational activation of C3 on surfaces (16, 53, 54). In human blood, C3 is the most abundant complement protein with a concentration of ~2 mg/mL, therefore, allowing the constant availability of a certain amount of hydrolyzed C3. In turn, the AP can directly be activated by the surface-bound C3 or hydrolyzed C3, as C3(H_2_O) adopts a structure similar to C3b allowing binding with fB to make a convertase initiating the AP (55). In the presence of high density IgG bound to the target antigen, the immune complexes can bind the C3b formed from cleavage of the hydrolyzed C3 and form a convertase, or form convertases from activation of C3 on the meningococcal surfaces directly (56). Moreover, Rawal and Pangburn showed that the CP C3 convertase C4bC2a is able to cleave C5, but has a lesser affinity for substrate C5 than C3 (57). Despite the low turnover of C5, in conditions with high IgG binding to target antigen, which cleaves C2 and C4 to initiate CP, it is sufficient to generate physiological amounts of MAC. In the clinic, patients with primary C3 deficiencies are at an early age highly susceptible to pyogenic infections, especially *Streptococcus pneumoniae.* However, a few reports show that these effects subside in adulthood with the development of the adaptive immune system and a higher acquisition of immunoglobulin (55, 58, 59).

An essential serine protease for the activation of the LP is MASP-2. Currently, anti-MASP-2 inhibitors such as Narsoplimab (OMS721) from Omeros is being evaluated as an alternative candidate for the treatment of aHUS. It has had success in the clinic against IgA vasculitis-associated nephritis and thrombotic microangiopathies (42, 60). However, the role of LP in immunity against meningococci has not been clearly defined. Here, SBA experiments with the anti-MASP-2 mAb showed no implication in meningococcal clearance in either pre or post-vaccine sera, indicating that LP may not be essential.

Limitations of the present investigation include that for logistical reasons, we had no access to sera from PNH or aHUS patients immunized with Menveo. Moreover, we did not include results of our analyses with serogroup B isolates (manuscript in preparation) as serogroup B vaccines are multi-protein based, analysis of the immune response is complex and inclusion of data with a serogroup B vaccine would have gone beyond the scope of the present manuscript. Despite the fact that our sample group was relatively small, the sera tested comprised a suitable range of antibody titers to draw firm conclusions.

In summary, our results highlight the advantage of AP inhibitors over C3 and C5 inhibitors as complement therapeutics, as they do not completely prevent terminal pathway activation and meningococcal killing by capsule specific antibodies. Treatment with AP inhibitors should therefore be combined with meningococcal vaccination. In aHUS patients, Gäckler et al. found that one vaccination with an ACWY conjugate vaccine is not sufficient to elicit antibody responses against all four serogroups in the majority of patients (61). A re-vaccination may therefore be advisable.

## Data Availability Statement

The original contributions presented in the study are included in the article/**Supplementary Material**. Further inquiries can be directed to the corresponding author.

## Ethics Statement

Ethical approval was obtained from the Ethical Committee of Northwest and Central Switzerland (Ethikkommission Nordwest- und Zentralschweiz (EKNZ), Studie 2018-02341). The patients/participants provided their written informed consent to participate in this study.

## Author Contributions

EI and LM contributed to the study conceptualization, data acquisition, analysis and data interpretation and drafted the manuscript. GP contributed to study conceptualization and design, data analysis and interpretation and revised the manuscript. AS, CT, NZ, TH, and MK contributed to study conceptualization and design and revised the manuscript. All authors contributed to the article and approved the submitted version.

## Conflict of Interest

GP received funding to conduct the study under a research agreement contract between Novartis Pharma AG and the Swiss Tropical and Public Health Institute. The funders initiated study design and decision to implement the SBA assay and compare activity of the tested drugs. The funders had no role in data collection and analysis. GP has received a research grant from Novartis Pharma AG. AS, CT, NZ, TH, and MK are full-time employees of Novartis.

The remaining authors declare that the research was conducted in the absence of any commercial or financial relationships that could be construed as a potential conflict of interest.

## Publisher’s Note

All claims expressed in this article are solely those of the authors and do not necessarily represent those of their affiliated organizations, or those of the publisher, the editors and the reviewers. Any product that may be evaluated in this article, or claim that may be made by its manufacturer, is not guaranteed or endorsed by the publisher.
