# Supplementary material for: Alternative Complement Pathway Inhibition Does Not Abrogate Meningococcal Killing by Serum of Vaccinated Individuals
Source: Front Immunol. 2021 Oct 8;12:747594. doi: 10.3389/fimmu.2021.747594 (PMC8531814; doi:10.3389/fimmu.2021.747594)
Supplement: Supplementary file 1 [file DataSheet_1.pdf]

Supplementary Material

Supplementary Data

Supplementary Table 1. Summary of the serogroup-specific IgG and IgM concentrations in human serum samples from subjects vaccinated with the MenACWY-CRM197 conjugate vaccine Menveo. The concentration of polysaccharide specific IgG and IgM in the serum samples were determined using the CDC 1992 reference serum with known concentration of IgG, IgM and IgA towards the different polysaccharides. Abbreviations: Pre = pre vaccination serum sample; 2 weeks (w) and 2 months (m) after vaccination with Menveo.

	**Subject**	2			3			9			11			12		
	**Serum sample**	Pre	w	m	Pre	w	m	Pre	w	m	Pre	w	m	Pre	w	m
Polysaccharide specific IgG (µg/mL)	A	49	97.7	93	12.6	22.4	28.8	0	39.1	23.4	0	24.9	13.6	1.6	66.5	38.6
	C	4.6	20.6	21.1	1.2	3.5	3.2	3	29.9	24.6	0.7	18.5	10.5	4.7	26.4	24.2
	W	5.2	22.1	19	3.2	3.7	4.2	7.7	9.1	8.4	2.4	5.2	6.6	7	26	25
	Y	9.9	13.7	10.6	11.3	18.5	17.1	9.9	34.9	30.5	3.4	5.3	4.3	19.8	43.5	42.5
Polysaccharide specific IgM (µg/mL)	A	7.4	23.6	26.7	1.7	0	0	0	20.4	8.6	5.9	17	13.8	5.4	20.9	17.6
	C	0.6	0.4	0.4	0.3	0.2	0.2	0.5	4.8	2.6	0.5	3.5	2.7	0.5	2	1.2
	W	0.6	1.1	0.8	0.5	0.2	0.2	1.8	2	2.1	0.8	1.1	1.1	1.1	1.8	1.9
	Y	1.5	1.4	1.5	2.1	0.7	0.7	1.9	4.4	3.8	1	2.5	1.2	1.1	2.4	1.9

Supplementary Table 2. Summary of the average SBA titers of the human serum samples of subjects vaccinated with the MenACWY-CRM_{197} vaccine Menveo. SBA titers are the reciprocal dilutions of serum that result in 50% killing of the bacteria after 60 minutes incubation. A lack of SBA is indicated with the number 2.5. The lowest serum dilution tested was 1:5. Abbreviations: Pre = pre vaccination serum sample; 2 weeks (w) and 2 months (m) after vaccination with Menveo.

	**Subject**	2			3			9			11			12		
	**Serum sample**	Pre	w	m	Pre	w	m	Pre	w	m	Pre	w	m	Pre	w	m
Mean SBA titer	A 2602	22	26	31	23	27	23	2.5	26	24	2.5	7	8	2.5	70	108
	C 4207	86	35	39	2.5	2.5	2.5	21	55	58	2.5	31	24	101	120	128
	W 1682	26	105	112	2.5	21	24	2.5	2.5	2.5	12	37	27	25	172	126
	Y 5810	14	113	98	7	27	33	8	81	64	7	8	15	30	123	83

Supplementary Table 3. Changes in serum bactericidal activity for the different serum/serogroup combinations tested.

**Subject**		2	3	9	11	12	n
**Change in SBA**	Negative to positive		W	A	A C	A	5
	Increase	A W Y	Y	C Y	W Y	W Y	10
	No change		A			C	2
	Decrease	C					1
	Remained negative		C	W			2

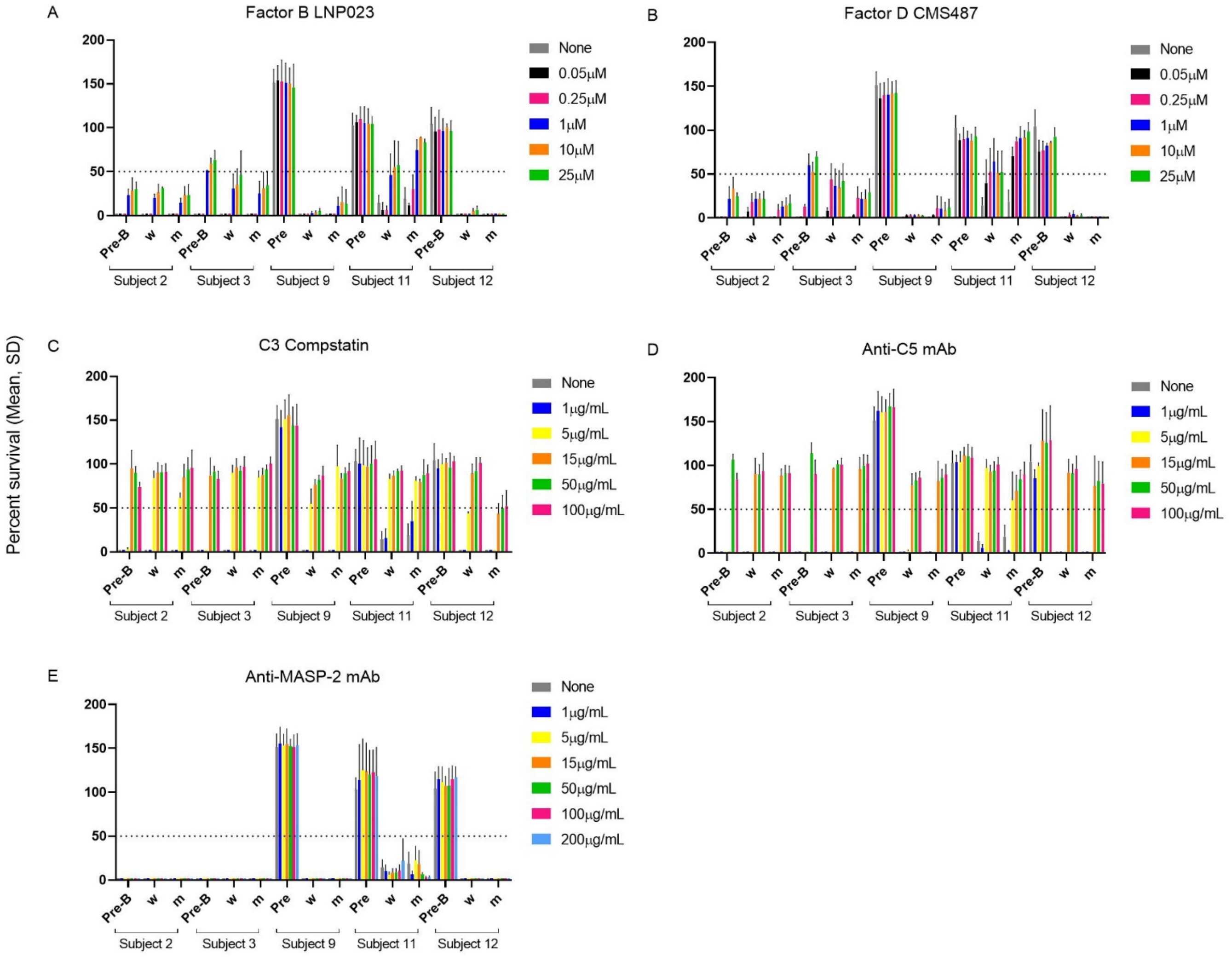

Supplementary Figure 1. The effect of complement inhibitors on the SBA titers measured against the meningococcal serogroup A 2602 case isolate. All subjects were vaccinated with the tetravalent meningococcal polysaccharide conjugate vaccine Menveo (MenACWY-CRM_{197}). All sera samples for each subject were diluted 1:5 and assayed with the internal complement. The horizontal dotted line represents 50% killing of bacteria after 60 min incubation. For each graph, grey bars are test sera without added inhibitors. Data for each inhibitor are triplicate technical replicates. SD: standard deviation of mean. Abbreviations: Pre = pre vaccination serum sample; 2 weeks (w) and 2 months (m) after vaccination with Menveo.

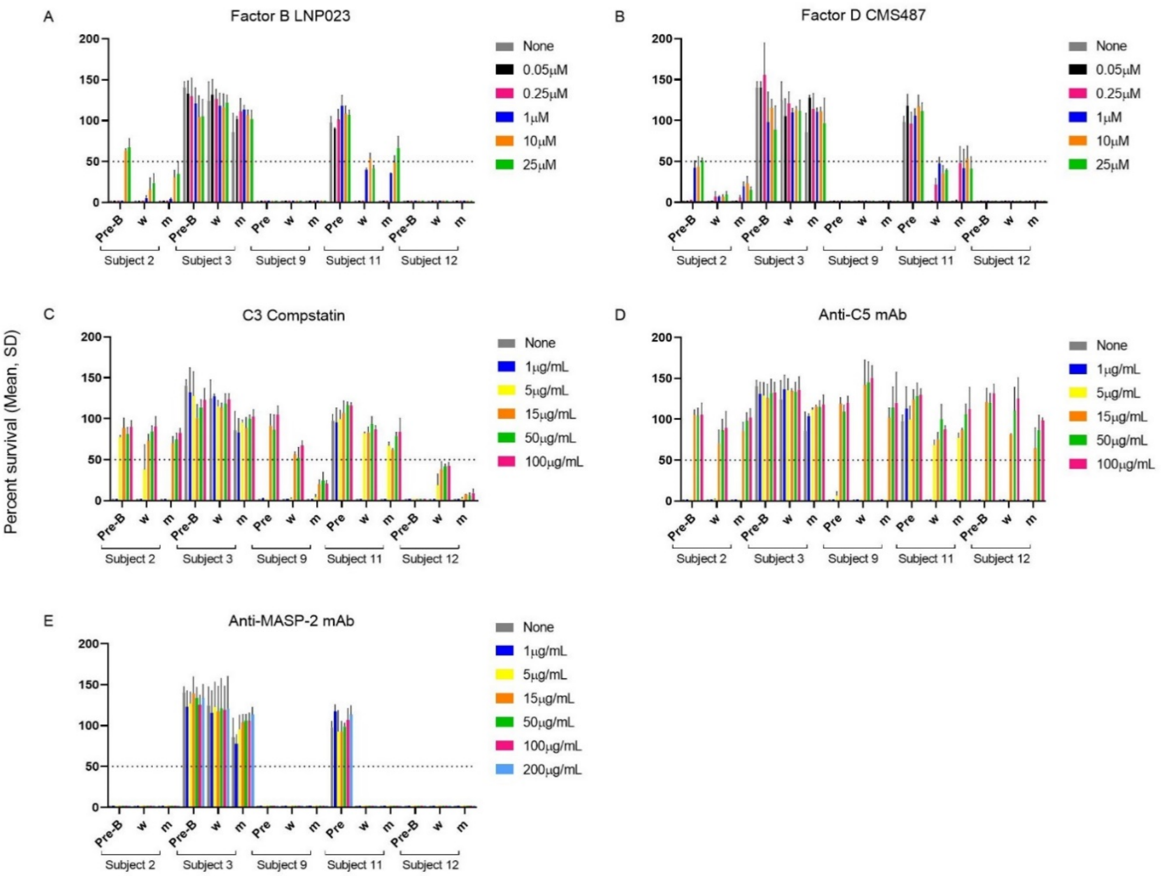

Supplementary Figure 2. The effect of complement inhibitors on the SBA titer measured against the meningococcal serogroup C 4207 case isolate. All subjects were vaccinated with the meningococcal polysaccharide conjugate vaccine Menveo (MenACWY-CRM$_{197}$). All sera samples for each subject were diluted 1:5 and assayed with the internal complement. The horizontal dotted line represents 50% killing of bacteria after 60 min incubation. For each graph, grey bars are test sera without added inhibitors. Data for each inhibitor are triplicate technical replicates. SD: standard deviation of mean. Abbreviations: Pre = pre vaccination serum sample; 2 weeks (w) and 2 months (m) after vaccination with Menveo.

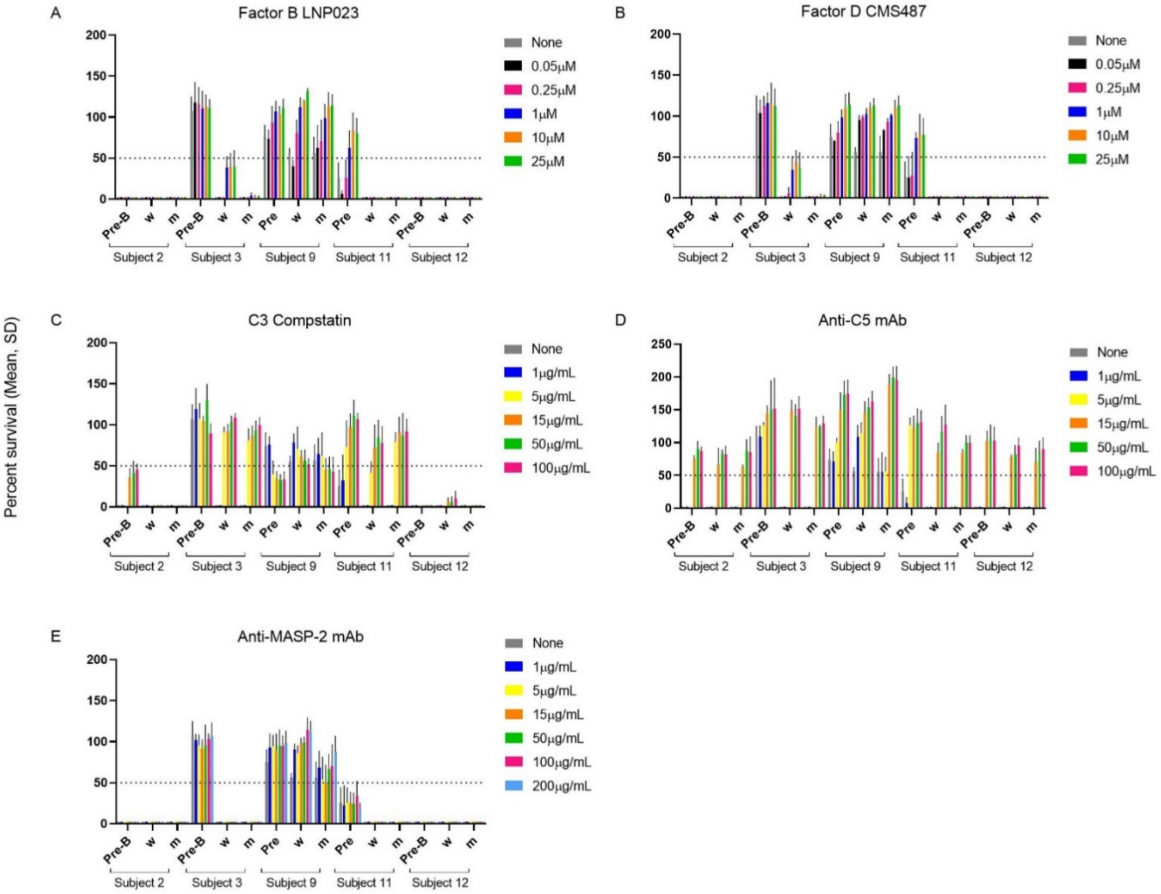

Supplementary Figure 3. The effect of complement inhibitors on the survival of the meningococcal serogroup W 1682 case isolate in pre- and post- Menveo vaccination sera.

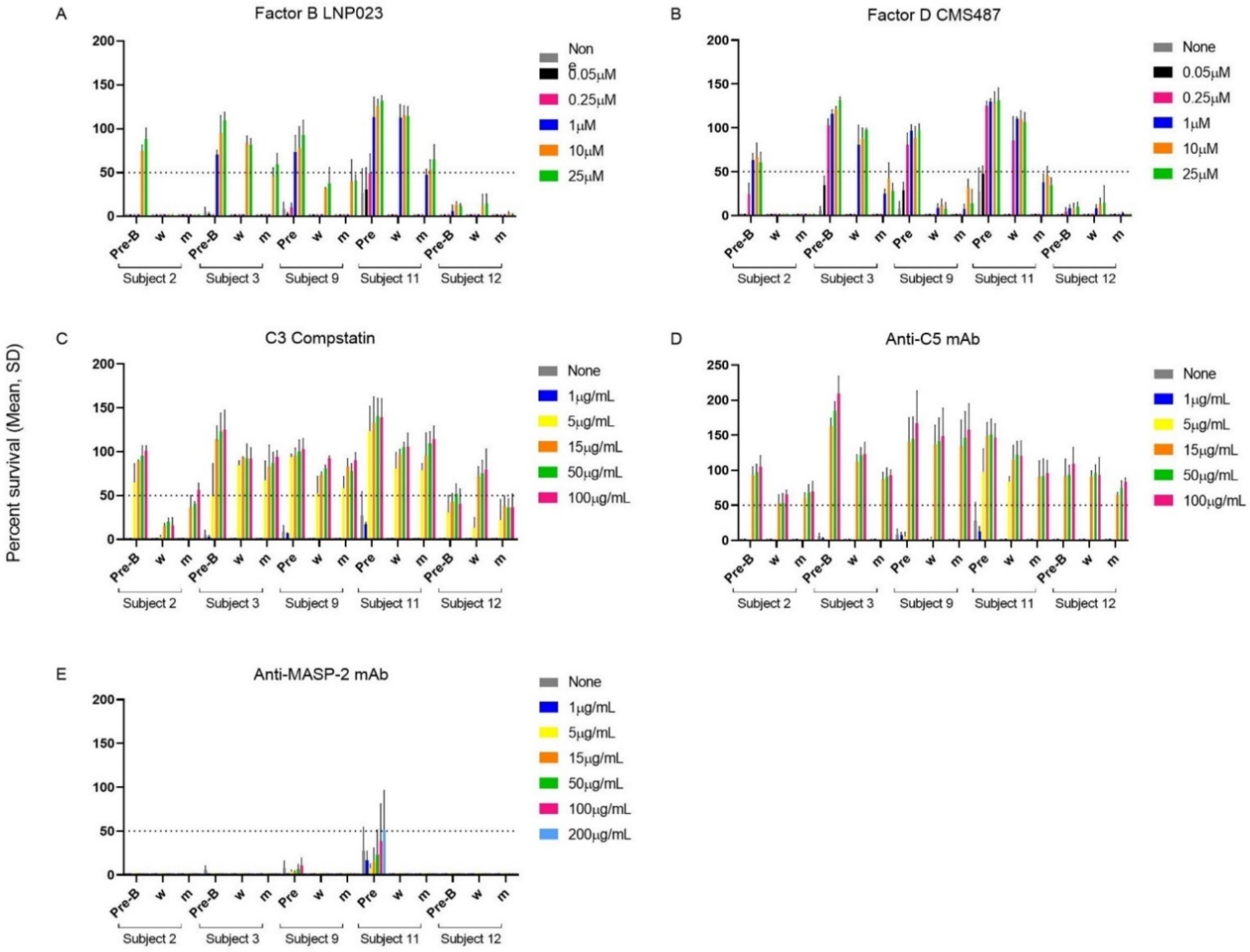

Supplementary Figure 4. The effect of complement inhibitors on the survival of the meningococcal serogroup Y 5810 case isolate in pre- and post- Menveo vaccination sera.

frontiers
